# Comparing different approaches for operationalizing subjective cognitive decline: impact on syndromic and biomarker profiles

**DOI:** 10.1038/s41598-021-83428-1

**Published:** 2021-02-23

**Authors:** Patricia Diaz-Galvan, Daniel Ferreira, Nira Cedres, Farshad Falahati, Juan Andrés Hernández-Cabrera, David Ames, Jose Barroso, Eric Westman

**Affiliations:** 1 Department of Clinical Psychology, Psychobiology, and Methodology, Faculty of Psychology and Speech Therapy, University of La Laguna, La Laguna, Tenerife, Spain; 2 Division of Clinical Geriatrics, Center for Alzheimer Research, Department of Neurobiology, Care Sciences and Society, Karolinska Institutet, Stockholm, Sweden; 3 Academic Unit for Psychiatry of Old Age (St. Vincent’s Health), University of Melbourne, Kew, VIC Australia; 4 National Ageing Research Institute, Parkville, VIC Australia; 5 Department of Neuroimaging, Center for Neuroimaging Sciences, Institute of Psychiatry, Psychology and Neuroscience, King’s College London, London, UK

**Keywords:** Alzheimer's disease, Dementia, Magnetic resonance imaging, Cognitive ageing

## Abstract

Subjective cognitive decline (SCD) has been proposed as a risk factor for future cognitive decline and dementia. Given the heterogeneity of SCD and the lack of consensus about how to classify this condition, different operationalization approaches still need to be compared. In this study, we used the same sample of individuals to compare  different SCD operationalization approaches. We included 399 cognitively healthy individuals from a community-based cohort. SCD was assessed through nine questions about memory and non-memory subjective complaints. We applied four approaches to operationalize SCD: two hypothesis-driven approaches and two data-driven approaches. We characterized the resulting groups from each operationalization approach using multivariate methods on comprehensive demographic, clinical, cognitive, and neuroimaging data. We identified two main phenotypes: an amnestic phenotype characterized by an Alzheimer’s Disease (AD) signature pattern of brain atrophy; and an anomic phenotype, which was mainly related to cerebrovascular pathology. Furthermore, language complaints other than naming helped to identify a subgroup with subclinical cognitive impairment and difficulties in activities of daily living. This subgroup also showed an AD signature pattern of atrophy. The identification of SCD phenotypes, characterized by different syndromic and biomarker profiles, varies depending on the operationalization approach used. In this study we discuss how these findings may be used in clinical practice and research.

## Introduction

In 2014, the subjective cognitive decline initiative (SCD-I) published a research framework for SCD as a risk factor for mild cognitive impairment (MCI) and Alzheimer’s disease (AD)^1^. However, SCD is an heterogeneous clinical condition that can be related to other pathologies such as cerebrovascular disease^2^. Despite intense research, the field still lacks data on which is the best way to operationalize SCD, and the SCD-I has recently called for studies that compare different operationalization approaches of SCD^3^.

Since SCD is postulated as the pre-MCI stage^4^, hypothesis-driven approaches based on well-established MCI criteria could be useful to operationalize SCD. Alternatively, data-driven approaches may also be of interest. An option is operationalizing SCD subtypes based on the frequency and distribution of cognitive complaints. For instance, memory and word-finding complaints are frequently reported^5–7^. Other options are methodologically more complex. An example is the study by Amariglio et al.^8^, in which the authors applied regression models to identify the specific complaints associated with lower cognitive performance on cross-sectional data.

Reaching a consensus on how to operationalize SCD is important because different operationalization approaches may provide groups with different syndromic and biomarker profiles^9^. To our knowledge, there are no studies comparing different operationalization approaches in the same sample of SCD individuals. Hence, our aims were to: (1) apply four different SCD operationalization approaches in the same sample; (2) describe the frequency of subtypes resulting from the different operationalization approaches; (3) compare the approaches and subtypes in terms of cognitive, clinical, and structural magnetic resonance imaging (sMRI) biomarker profiles. We hypothesized that memory and word-finding complaints would be frequently reported, hence subtypes based on memory and word-finding complaints would have a high frequency. Although objective cognitive impairment was not expected, congruent with the definition of SCD, we anticipated lower cognition (subclinical impairment) and abnormal sMRI biomarkers in SCD individuals, with different profiles depending on the operationalization approach. Reaching a definitive answer on which is the best operationalization of SCD may only be achieved by large multi-center studies investigating different cohorts and using various instruments for measuring subjective complaints. We thus consider our current study as a first step towards providing preliminary data and methodological examples that may guide and encourage future studies in this area.

## Methods

### Participants

A total of 399 individuals from the GENIC-database^10, 11^ were included in this study. The GENIC is a prospective community-based study from the Canary Islands, Spain. Details on this cohort are provided in previous publications^11^. Briefly, recruitment was carried out through primary care health centers, advertisements in local schools, and relatives and acquaintances of the research staff. For the current study, individuals were selected according to the basic criteria from the research framework for SCD^1^: (a) normal age-, gender-, and education-adjusted performance on extensive neuropsychological testing according to clinical normative data; (b) normal performance in activities of daily living and global cognition defined in this study by a score ≤ 4 on the Blessed Dementia Rating Scale (BDRS)^12^, a score ≤ 5 on the Functional Activity Questionnaire (FAQ)^13^, and a score ≥ 26 on the Mini-Mental State Examination (MMSE)^14^; (c) absence of MCI or dementia; (d) and absence of past or present psychiatric or neurologic diseases, medical disorders, substance abuse, or use of medications that might explain the presence of subjective cognitive complaints.

This study was approved by the Ethical Committee on Research and Animal Welfare from the University of La Laguna, Spain. Each participant provided written informed consent and completed the same experiment protocol in accordance to the Declaration of Helsinki.

### Subjective cognitive complaints

Subjective complaints were assessed through nine yes/no-type questions covering the following cognitive domains: memory, orientation, executive functions, face recognition, language production, language comprehension, word-finding, reading, and writing (Table 1).

**Table 1:** Questions to assess subjective cognitive complaints in the GENIC cohort.

Cognitive domain	Question
*Orientation*	1. Do you find it harder to orient yourself in time or space?
*Memory*	2. Do you have memory problems?
*Visuoperception*	3. Do you find it harder to recognize familiar faces or people you do not see often?
*Executive functions*	4. Do you find it harder to manage money or do mental arithmetic?
*Language*	5. Do you find it hard to find words? 6. Do you have any problems with reading? 7. Do you have any problems with writing? 8. Have you noticed whether you speak less or worse lately? 9. Do you find it harder to follow a conversation? Do you find it harder to understand what people say to you?

The questions refer to changes in approximately the last six months and are coded as 0 (absence of complaint) or 1 (presence of complaint). We calculated the total amount of complaints by adding the scores for each question, ranging from 0 (no complaints) to 9 (maximum number of complaints).

In a simplified manner, these nine questions cover the five cognitive domains included in validated scales such as Everyday Cognition (ECog)^15^ . Furthermore, these nine questions also extend the domains covered by the only questionnaire validated in Spanish, i.e., Subjective Cognitive Decline-Questionnaire (SCD-Q)^16^. Item-by-item correspondence among these three methods can be seen in Table 2. Of note, both ECog and SCD-Q are endorsed by the SCD-I, and ECog is the most commonly used scale among the participating studies of the world-leading SCD initiative^5^. Answers were referred to cognitive changes observed during approximately the last six months. Each answer was scored as one (presence) or zero (absence). A total score was computed by adding up all the scores for each complaint, giving a continuous variable ranging from 0 to 9, where higher scores indicate greater number of complaints.

**Table 2:** Item-by-item cognitive domain correspondence among GENIC, eCog, and MyCog Questionnaires for SCD.

GENIC	Ecog	SCD-Q (MyCog)
Orientation		
1. Do you find it harder to orient yourself in time or space?	Following a map to find a new location Reading a map and helping with directions when someone else is driving Finding his/her car in a parking lot Finding the way back to a meeting spot in the shopping mall or other location Finding his/her way around a familiar neighborhood Finding his/her way around a familiar store Finding his/her way around a house visited many times	*NON ASSESED*
Memory		
2. Do you have any memory problems (do you find it harder to remember what you have read, where you have placed objects, important appointments, what you wanted to do, what you did yesterday)?	Remembering a few shopping items without a list Remembering things that happened recently (such as recent outings, events in the news) Recalling conversations a few days later Remembering where she/he has placed objects Repeating stories and/or questions Remembering the current date or day of the week Remembering he/she has already told someone something Remembering appointments, meetings, or engagements	I’m worse at recalling the details of a recent family event I find it harder to remember the result of a recent sporting event I find it harder to remember the details of a conversation I find it harder to remember things without using strategies (lists, diary, etc.) I find it harder to remember the details of recent new I find it harder to remember famous people’s names I find it harder to remember the names of people I’ve met recently I find it harder to remember street and city names - I find it harder to describe the plots of films
Visuoperception		
3. Do you find it harder to recognize familiar faces or people you do not see often?	*NON ASSESED*	*NON ASSESED*
Attention / Executive Functions		
4. Do you find it harder to manage money or do mental arithmetic?	The ability to do two things at once Returning to a task after being interrupted The ability to concentrate on a task without being distracted by external things in the environment Cooking or working and talking at the same time Planning the sequence of stops on a shopping trip The ability to anticipate weather changes and plan accordingly Developing a schedule in advance of anticipated events Thinking ahead Thinking things through before acting Keeping living and work space organized Balancing the checkbook without error Keeping financial records organized Prioritizing tasks by importance Using an organized strategy to manage a medication schedule Keeping mail and papers organized	I find it harder to learn new telephone numbers I find it harder to find personal possessions (keys, telephone, utensils, etc.) I find it harder to remember doctor’s appointments I find it harder to concentrate on what I am doing I find it harder to remember the names of places I’ve visited recently I’m worse at planning things that aren’t part of my daily routine (travel, excursions, etc.) I find it harder to use electronic devices I find it harder to start new or different things Finds it harder to start conversations I find it harder to do mental arithmetic I find it harder to do more than one thing at once without getting agitated I find it harder to remember sums of money (payments or debts)
Language		
5. Do you find it harder to find words? 6. Do you have any problems with reading? 7. Do you have any problems with writing? 8. Have you noticed whether you speak less or worse lately? 9. Do you find it harder to follow a conversation? Do you find it harder to understand what people say to you?	Verbally giving instructions to others Following a story in a book Understanding the point of what other people are trying to say Describing a program he/she has watched on television Understanding spoken directions or instructions Forgetting the names of objects Finding the right words to use in a conversation Remembering the meaning of common words Communicating thoughts in a conversation	I find it harder to follow the plot of a book I’m worse at finding the word I want to use in a conversation I find it harder to understand things the first time someone says them

### SCD operationalization approaches

Individuals were classified as healthy controls (HC) if they did not endorse any subjective cognitive complaint, or as SCD if they endorsed one or more complaints. We applied four operationalization approaches on the SCD individuals as follows:

1. A hypothesis-driven approach based on Winblad’s criteria for MCI (from here, ***Clinical approach***)^17^. The so-called Winblad’s criteria rely on a clinical judgment for determining cognitive impairment in MCI and include four subtypes: amnestic single or multiple domain and non-amnestic single or multiple domain^17^. We identified the same cognitive subtypes on SCD individuals based on the type of subjective complaint instead of on actual objective impairment. This approach gave four SCD subtypes depending on whether individuals reported a complaint in memory alone (*amnestic single-domain SCD,* aSCD-sd); in memory and other cognitive domains (*amnestic multiple-domains SCD*, aSCD-md); in a cognitive domain alone other than memory (*non-amnestic single-domain SCD,* naSCD-sd); or more than one cognitive domain other than memory (*non-amnestic multiple-domains SCD,* naSCD-md).
2. A hypothesis-driven approach based on the MCI criteria from Mayo Clinic (US), which puts greater emphasis on the -1.5 SD criterion to define cognitive impairment (from here, ***Psychometric approach***)^18^. These criteria were adjusted to SCD by requiring a total number of complaints. Since the total number of complaints variable was not normally distributed (Fig. 1A), we chose the 90th percentile instead of the -1.5 SD to determine the cut-off for SCD, as suggested elsewhere^19^.

**Figure 1:**
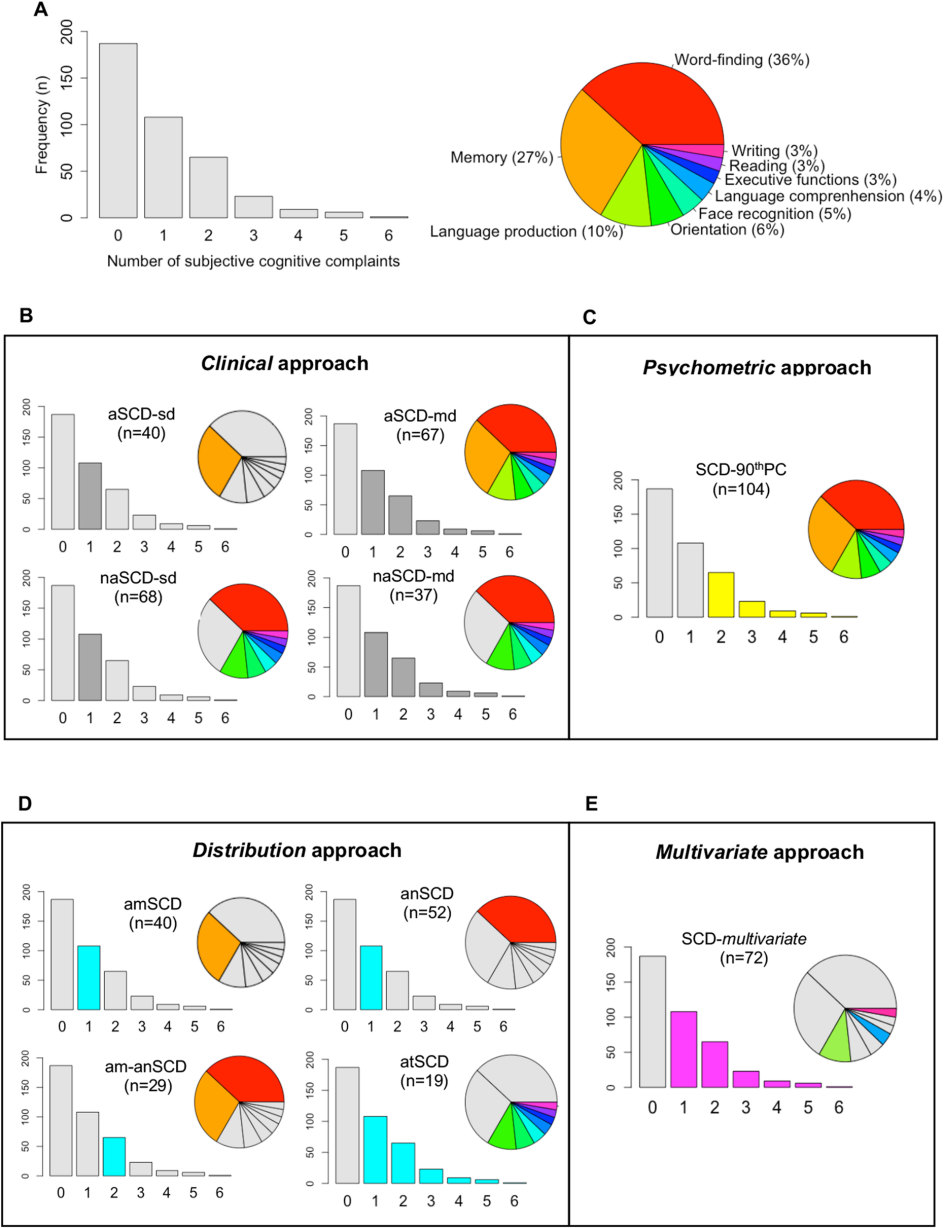
Overview of subjective cognitive complaints and SCD groups in the GENIC cohort. **(a)** Frequency of subjective cognitive complaints. (**b–e)** Overview of the SCD groups according to the four operationalization approaches. All the bar charts show number of subjective cognitive complaints in the x-axis and the frequency (n) in the y-axis. HC, healthy controls; aSCD-sd = amnestic Subjective Cognitive Decline—single domain; aSCD-md, amnestic Subjective Cognitive Decline—multiple domain; naSCD-sd, non-amnestic Subjective Cognitive Decline—single domain; naSCD-md, non-amnestic Subjective Cognitive Decline—multiple domain; SCD-90thPC, Subjective Cognitive Decline defined by the presence of two or more cognitive complaints, corresponding to the 90thPC of the total number of complaints variables; anSCD*,* anomic Subjective Cognitive Decline; amSCD*,* amnestic Subjective Cognitive Decline; am-anSCD*,* amnestic and anomic Subjective Cognitive Decline; atSCD*,* atypical Subjective Cognitive Decline; SCD-*multivariate*, Subjective Cognitive Decline defined by the presence of language production, language comprehension and/or writing complaint alone or in combination with other complaints.
3. A data-driven approach based on the frequency and distribution of subjective cognitive complaints observed in the sample (from here, ***Distribution approach***). We explored the frequencies of the different cognitive complaints when they were reported alone or in combination with the other complaints. For that, we divided the variable ‘number of complaints’ into quartiles; then, we investigated the distribution of the different complaints within each quartile in order to identify the SCD groups. We elaborate more on this approach in the results section: we describe the procedure that uncovered which specific complaints define the different SCD subtypes in our cohort; we also explain how individuals were assigned to the resulting subgroups.
4. A data-driven approach using multivariate data analysis (from here, ***Multivariate approach***). Amariglio et al.^8^ applied predictive models to identify which complaints were associated with lower cognitive performance in cross-sectional data from a large community-based cohort. The authors reported that the complaint of “getting lost” was strongly associated with lower cognitive performance. Inspired by this study, we also applied a predictive model to identify which complaints were associated with lower cognitive performance in our cross-sectional data. To increase the sensitivity of this approach towards early stages of neurodegenerative diseases, we aimed to identify complaints predicting lower performance in cognitive variables that are strongly associated with measures of activities of daily living (ADL). The reason behind this decision was to capture subclinical levels of impairment in cognition and ADL, which often prelude progression to MCI and dementia (stage III of preclinical AD)^1^. We applied a principal component analysis (PCA) on 67 cognitive variables and 4 ADL measures. The dimension that clustered cognitive measures together with the ADL measures was defined as our dimension of interest. We then conducted a predictive model (random forest regression model) to identify which complaints predicted such clinical-cognitive dimension of interest. We finally created the corresponding SCD group based on the identified complaints. Complementary, the PCA also reduced the dimensionality of the cognitive and clinical data (71 variables). The resulting components were used for further representation of certain results.

### Demographic, clinical, and cognitive variables

Age and sex were included as demographic variables. We used the Information subtest from the Wechsler Adult Intelligence Scale—Third edition (WAIS-III) as an estimation of crystallized intelligence/education^11,20^. Clinical measures included information on ADL from both FAQ^13^ and BDRS^12^ (total score as well as scores from the three BDRS subscales: 1) changes in performance on everyday activities, 2) habits, and 3) personality, interest, and drive). Depressive symptomatology was assessed with the Beck Depression Scale (BDI)^21^ in individuals below 63 years of age, and the Geriatric Depression Scale (GDS)^22^ in individuals 63 years old or older. Z-scores from both scales were calculated and combined together in order to have a single measure of depressive symptomatology^23^. We highlight that none of the participants in this study had a clinical diagnosis of depression or were taking antidepressant medication, and scores in these scales were within the normal range. We applied a comprehensive neuropsychological protocol including tests for processing speed, attention, executive functions, premotor functions, memory, visuoconstructive, visuoperceptive, and visuospatial functions, and language functions. The neuropsychological protocol includes 67 cognitive variables and is fully detailed in previous publications^24^.

### Magnetic resonance imaging (MRI) data acquisition and processing

Participants were scanned using a 3.0 T General Electric imaging system (Milwaukee, United States). A three-dimensional T1-weighted fast spoiled gradient echo (FSPGR) sequence was acquired in sagittal plane (repetition time/echo time/inversion time = 8.73/1.74/650 ms, field of view = 250 × 250 mm, matrix = 250 × 250 mm, flip angle = 12°, slice thickness = 1 mm).

The T1-weighted images were processed with FreeSurfer 5.1.0 (http://surfer.nmr.mgh.harvard.edu/) through our database system (theHiveDB)^25^ as detailed elsewhere^26^. Careful visual quality control was performed on both the original and the processed data, and manual edits were done when appropriate to ensure optimal output. Measures of thickness were calculated for 34 cortical regions from both hemispheres^27^, and measures of volume for 21 subcortical regions^28^. A measurement of total intracranial volume (ICV) was also estimated with FreeSurfer in order to account for individual differences in brain size on all the volumetric measures^29^.

### MRI biomarkers of AD and cerebrovascular disease

Previous research has linked SCD with AD and cerebrovascular disease^1,2,30,31^. AD-related neurodegeneration and cerebrovascular disease can be assessed in vivo with structural MRI. In the current study, we investigated how different SCD operationalization approaches may relate to sMRI biomarkers of AD-related neurodegeneration and cerebrovascular disease. A novel sMRI biomarker of AD is the “disease severity index”, which captures the AD signature atrophy pattern in SCD individuals^30^. This index is strongly associated with increased amyloid burden and higher risk of progression to MCI or dementia^31^. A common sMRI biomarker of cerebrovascular disease is white matter signal abnormalities (WMSA)^32,33^. Both methods are explained below.

The AD signature atrophy pattern (“disease severity index”) was calculated as in a previous publication^31^. Briefly, a classification model was trained on an external database to discriminate between 69 healthy controls and 39 AD dementia patients from the AIBL cohort (Australian Imaging Biomarkers and Lifestyle flagship study of ageing)^34^. The 34 measures of cortical thickness from both hemispheres and the 21 subcortical volumes from FreeSurfer were used as input data. These data were previously corrected for age and ICV, since both variables are known to influence brain morphology^29,35^. The variance in the sMRI measures related to age and ICV was estimated and removed from the original data using multiple linear regression. After correcting the data, the classification model was built using the orthogonal partial least square (OPLS) method included in the software package SIMCA (Sartorius Stedim AB, Umeå, Sweden). The OPLS method separates the systematic variation in the data into two blocks: predictive and orthogonal. The first component of the model is predictive and includes information related to class separation (e.g. AD vs. HC). The orthogonal components in the model, if any, are related to other variation in the data not related to the actual problem, such as within class variation. Each model receives an R^2^(X), an R^2^(Y), and a Q^2^(Y) value. R^2^(X) represents the explained variance between the criterion variable (Y) and predictor variables (X), for the predicted and the orthogonal components. R^2^(Y) represents the goodness of fit of the model and refers to the fraction of the criterion variable (Y) variation modeled in the component, using the predicted model. Q^2^(Y) defines how well the model predicts new data. The significance of a model is based on the Q^2^(Y) parameter and is reported as acceptable (Q^2^ > 0.1), good (Q^2^ > 0.5), and optimal (Q^2^ > 0.9)^36^. In the current study, the sevenfold validation method to separate AD patients from HC achieved an R^2^(X) value of 0.171, an R^2^(Y) value of 0.848, and a Q^2^(Y) value of 0.700, indicating a high performance to discriminate between the HC and AD groups. The brain regions that contributed the most to this model were the hippocampal volume, the precuneus, the right supramarginal gyrus, and the inferior parietal gyrus, all of them displaying reduced values in the AD group. The inferior part of the lateral ventricles was also important, displaying larger volume in the AD group, as in previous publications^31^. Afterwards, age- and ICV-corrected values of the same cortical and subcortical sMRI variables from the GENIC individuals were projected onto this classification model as unseen data. By doing this, all the GENIC individuals receive a score for the “disease severity index”. This score reflects the AD signature atrophy pattern and ranges from 0 to 1. Values close to 0 are indicative of a HC-like pattern, and values close to 1 reflect an AD-like pattern of brain atrophy. This index can be used either as a continuous variable, or as a dichotomous variable by applying a threshold.

The WMSA volume was calculated on T1-weighted images using FreeSurfer, subsequently extended to label white matter lesions^28^. WMSA is an indicator of underlying cerebrovascular disease. This procedure has demonstrated sensitivity in measuring white matter damage in both healthy and SCD individuals, as well as in patients with AD^23,37,38^. The T1-weighted WMSA volume from FreeSurfer is correlated with hyperintensity volume measured on T2/FLAIR, as well as with microstructural white matter changes as measured on diffusion tensor imaging data^10,38,39^.

### Statistical analysis

Two per cent of the values were missing across all cognitive variables and were thus imputed for subsequent analyses. Random forest analyses (5000 trees) were conducted to characterize the SCD groups against the HC across multiple variables (classification models) or to investigate the association between multiple predictors and an outcome variable (regression models), while avoiding multiple testing. In random forest models, the contribution of the predictors in the models is reported as *Imp* (importance), which reflects the relative error in prediction when a predictor is excluded from the model. Pearson, point-biserial, and partial correlations were performed to study relationships between variables. ANOVA/ANCOVA, Mann–Whitney, and Kruskal Wallis tests were conducted to investigate between-group differences in continuous variables and the Chi-square test was used for categorical variables. Posthoc analyses were conducted in ANOVA/ANCOVA for the sMRI variables using the Hochberg correction for multiple comparisons^40^. Principal component analysis (PCA) was conducted on the 67 cognitive variables and 4 ADL variables for reducing data dimensionality and identifying a clinical-cognitive dimension of interest as explained above in the “SCD operationalization approaches” section. The components from this PCA were also used for investigating borderline cognitive performance in a reduced set of cognitive variables. Borderline cognitive performance was defined as a score below the 10th percentile on the cognitive components after adjusting for age, sex, and crystallized intelligence/education (WAIS-III Information subtest) using multiple linear regression analysis. A *p *value ≤ 0.05 (two-tailed) was deemed significant in all these analyses.

## Results

### Frequency of subjective cognitive complaints and SCD groups

Overall, 53.1% of the individuals (n = 212) reported at least one complaint. Word-finding (36%, n = 145) and memory (27%, n = 107) were the most frequent complaints (Fig. 1A). Table 3 shows the main demographic and clinical characteristics of the different SCD groups by operationalization approach. Additionally, the association of each cognitive complaint with demographic, clinical, cognitive, and MRI data are displayed on the supplementary Table S1.

**Table 3:** Characteristics of the SCD groups and healthy controls.

	HC	*Clinical *approach					*Psychometric* approach		*Distribution* approach					*Multivariate* approach	
		aSCD-sd	aSCD-md	naSCD-sd	naSCD-md	*p*	SCD-90thPC	*p*	amSCD	anSCD	am-anSCD	atSCD	*p*	*SCD-*multivariate	*p*
Count, n (%)^a^	187 (47)	40 (10)	67 (17)	68 (17)	37 (9)	< 0.001	104 (26)	−	40 (12)	52 (16)	29 (9)	19 (6)	< 0.001	59 (14)	-
Age, y	55.66 (11.3)	57.38 (11.5)	61.63 (11.9)	59.28 (10.7)^b^	63.73 (9.8)	< 0.001	62.37 (11.2)	< 0.001	57.38 (11.5)	60.02 (10.4)	58.59 (10.6)	57.05 (12.0)	0.087	61.93 (11.9)	< 0.001
Sex, % females	47	60	60	60	70	< 0.001	63	< 0.001	60	60	55	63	< 0.001	67	< 0.001
WAIS-III Information subtest	16.27 (6.3)	14.73 (6.1)	14.76 (6.3)	14.92 (5.9)	15.54 (6.3)	0.296	15.04 (6.3)	0.111	14.73 (6.1)	15.14 (5.7)	16.35 (6.3)	13.84 (12.0)	0.294	14.49 (6.3)	0.060
MMSE	28.73 (1.3)	28.95 (1.2)	28.40 (1.2)	28.68 (1.2)	28.57 (1.2)	0.227	28.46 (1.2)	0.030	28.95 (1.2)	28.61 (1.2)	26.90 (0.9)	28.89 (1.2)	0.686	28.43 (1.3)	0.011
FAQ	0.28 (0.7)	0.30 (0.6)	0.58 (1.1)	0.31 (0.7)	0.30 (0.7)	0.068	0.28 (1.0)	0.092	0.30 (0.6)	0.32 (0.7)	0.14 (0.4)	0.42 (0.1)	0.849	0.54 (1.0)	0.024
BDRS	0.43 (0.8)	0.51 (0.8)	1.03 (1.1)^b^	0.65 (0.9)	0.92 (1.1)^b^	< 0.001	0.99 (1.1)	< 0.001	0.51 (0.8)	0.66 (0.9)	0.62 (1.0)	0.84 (1.0)	0.098	1.09 (1.1)	< 0.001
Depressive sympt	−0.54 (−1.3–3.0)	−0.10 (−1.3–2.1)^b^	0.34 (−1.0–3.9)^b^	−0.10 (−1.3–3.0)^b^	0.34 (−1.0–3.9)^b^	< 0.001	0.34 (−1.0–3.9)	< 0.001	−0.10 (−1.3–2.1)^b^	-0.10 (-1.3–3.0)^b^	0.34 (-0.8–2.1)^b^	-0.10 (-1.0–3.5)^b^	< 0.001	0.34 (-1.0–3.9)	< 0.001
AD signature atrophy, (0–1)^c,d^	−0.01 (0.2)	0.14 (0.2)^b^	0.06 (0.2)^b^	0.14 (0.2)	0.11 (0.2)^b^	< 0.001	0.07 (0.2)	0.016	0.14 (0.2)^b^	0.11 (0.2)	0.04 (0.2)	0.23 (0.2)^b^	< 0.001	0.12 (0.2)	< 0.001
AD signature atrophy, % AD-like^c,d^	2	33	13	6	21	−	12	−	33	9	6	23	-	15	-
WMSA^c^	2621.3 (1207.0)	2820.8 (982.9)	3005.2 (2083.1)^b^	3185.1 (2011.1)	2852.8 (2151.9)	0.486	2949.5 (2088.7)	0.354	2820.8 (982.9)	3070.6 (1913.5)^e^	3023.0 (2353.9)	3498.6 (2220.6)	0.289	2874.2 (1580.9)	0.456

Values are reported as mean (SD) except for depressive symptomatology, where median (minimum and maximum values) are reported. All the analyses in this table were conducted to compare SCD groups versus HC, except for Chi-squared analyses in Count and Sex variables. ^a^The χ^2^ test was used for investigating between-group differences among the SCD subtypes within the *Clinical* and *Distribution* approaches (HC not included in these analyses. *Post-hoc* contrasts—naSCD-sd vs. aSCD-sd: χ^2^(1) = 7.26, *p* = 0.007; naSCD-sd vs. naSCD-md: χ^2^(1) = 9.15, *p* = 0.002; aSCD-md vs. aSCD-sd: χ^2^(1) = 6.81, *p* = 0.009; aSCD-md vs. naSCD-md: χ^2^(1) = 8.65, *p* = 0.003; anSCD vs. am-anSCD: χ^2^(1) = 6.53, *p* = 0.011; anSCD vs. atSCD: χ^2^(1) = 15.34, *p* < 0.001; amSCD vs- at SCD: χ^2^(1) = 7.47, *p* = 0.006.^b^Significant differences with HC ^c^n = 220. ^d^AD signature atrophy pattern determined by the predictive OPLS index which range from 0 (HC-like pattern of atrophy) to 1 (AD-like pattern of atrophy). Individuals were classified as AD-like when they obtained abnormal values in this index, corresponding to 0.32 according to the 90thPC. ^e^A trend for a significant WMSA increase was observed for the anSCD subtype (*p* = .074).

*HC* healthy controls, *SCD* Subjective Cognitive Decline, *aSCD-sd* amnestic Subjective Cognitive Decline-single domain, *aSCD-md* amnestic Subjective Cognitive Decline-multiple domain, *naSCD-sd* non-amnestic Subjective Cognitive Decline-single domain, *naSCD-md* non-amnestic Subjective Cognitive Decline-multiple domain, *SCD-90thPC* Subjective Cognitive Decline defined by the presence of two or more cognitive complaints, corresponding to the 90th PC of the total amount of cognitive complaints variable, *anSCD* anomic Subjective Cognitive Decline, *amSCD* amnestic Subjective Cognitive Decline, *am-anSCD* amnestic and anomic Subjective Cognitive Decline, *atSCD* atypical Subjective Cognitive Decline, *SCD-multivariate* Subjective Cognitive Decline defined by the presence of language production, language comprehension and/or writing complaints, alone or in combination with other complaints, *WAIS-III* Wechsler Adult Intelligence Scale-3rd Edition, *MMSE* Mini Mental State Examination, *FAQ* Functional Activity Questionnaire, *BDRS* Blessed Dementia Rating Scale, *sMRI* structural Magnetic Resonance Imaging, *AD* Alzheimer’s disease, *WMSA* white matter signal abnormalities.

1. *Clinical* approach (Table 3**,** Fig. 1B): the naSCD-sd (17%) and aSCD-md (17%) subtypes were significantly larger than the aSCD-sd (10%) and naSCD-md (9%) subtypes (*p* = 0.001).
2. *Psychometric* approach (Table 3, Fig. 1C)*:* since the total number of complaints variable was not normally distributed, the 90th percentile was chosen instead of the -1.5 SD to determine the cut-off for SCD, as suggested elsewhere^19^.
3. *Distribution* approach (Table 3, Fig. 1D)*:* Firstly, the variable ‘number of complaints’ was divided into quartiles. We observed that each quartile corresponded to zero (Q1), one (Q2), two (Q3), and three or more (Q4) cognitive complaints. Secondly, we scrutinized the distribution of complaints within each quartile. Since memory and word-finding complaints were the most frequently reported complaints, both dominated Q2, Q3, and Q4, either alone or in combination with other complaints. Therefore, for simplicity, we only illustrated the distribution of these two most common complaints. Based on this finding, four subtypes could be ascertained: *amnestic* SCD (amSCD; individuals with a complaint limited to memory); *anomic* SCD (anSCD; individuals with a complaint limited to word-finding); *amnestic and anomic* SCD (am-anSCD; individuals with two complaints limited to memory and word-finding); and *atypical* SCD (atSCD; individuals with one or more complaints in any cognitive domain other than memory or word-finding). Figure 2 shows the quartiles and the distribution of memory and word-finding complaints. The anSCD (16%) and amSCD (12%) subtypes were significantly larger than the atSCD (6%) subtype. The anSCD subtype was also larger than the am-anSCD subtype (9%) (*p* < 0.001).

**Figure 2:**
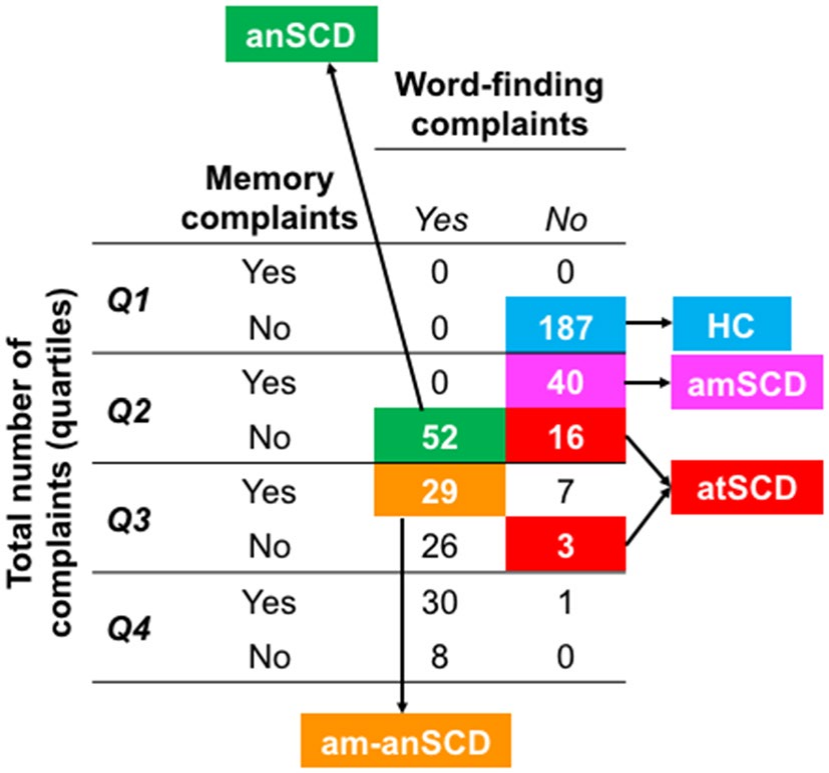
Identification of SCD subtypes in the *Distribution* approach. Cross-table of frequencies between each quartile of the variable ‘total number of complaints’ and the variables of ‘memory’ and ‘word-finding’ complaints. anSCD*,* anomic Subjective Cognitive Decline; amSCD*,* amnestic Subjective Cognitive Decline; am-anSCD*,* amnestic and anomic Subjective Cognitive Decline; atSCD, atypical Subjective Cognitive Decline.
4. *Multivariate* approach (Table 3, Fig. 1E): The PCA gave 5 components (R^2^ = 0.49). Components #1, #2, and #5 each explained 12% of the variance, and included variables related to visual functions, verbal episodic memory, and executive and premotor functions, respectively. Component #3 explained 8% of the variance and included variables related to visual memory. Component #4 explained 5% of the variance and clustered the ADL measures (i.e. FAQ and BDRS ‘changes in daily life activities’ subscale) together with several cognitive variables, including episodic memory, semantic fluency, and visual discrimination. Thus, component #4 was labeled as the ‘clinical severity component’ and it was considered as our component of interest for subsequent analyses in this approach. Lower scores in this component #4 indicates worse clinical and cognitive status. We then predicted component #4 by the nine different complaints as well as age, sex, WAIS-III Information subtest, and depressive symptomatology in a random forest model (regression). Component #4 was mainly predicted (R^2^ = 0.08) by the score on WAIS-III Information subtest (Imp = 0.13), the writing complaint (Imp = 0.05), and sex (Imp = 0.04). The language production complaint and language comprehension complaint marginally contributed to the prediction of component #4 (Imp < 0.01). Worse clinical-cognitive status as reflected by component #4 was associated with a lower score in WAIS-III Information (r = 0.28), the presence of complaints on writing (r_pb_ = -0.23), language production (r_pb_ = -0.03) and language comprehension (r_pb_ = -0.10), as well as female sex (r_pb_ = -0.04). Based on these results, individuals with a complaint either in writing, language comprehension, or language production, were classified as the SCD-multivariate group (14%), independently of whether those individuals also endorsed complaints in other domains.

**Figure 3:**
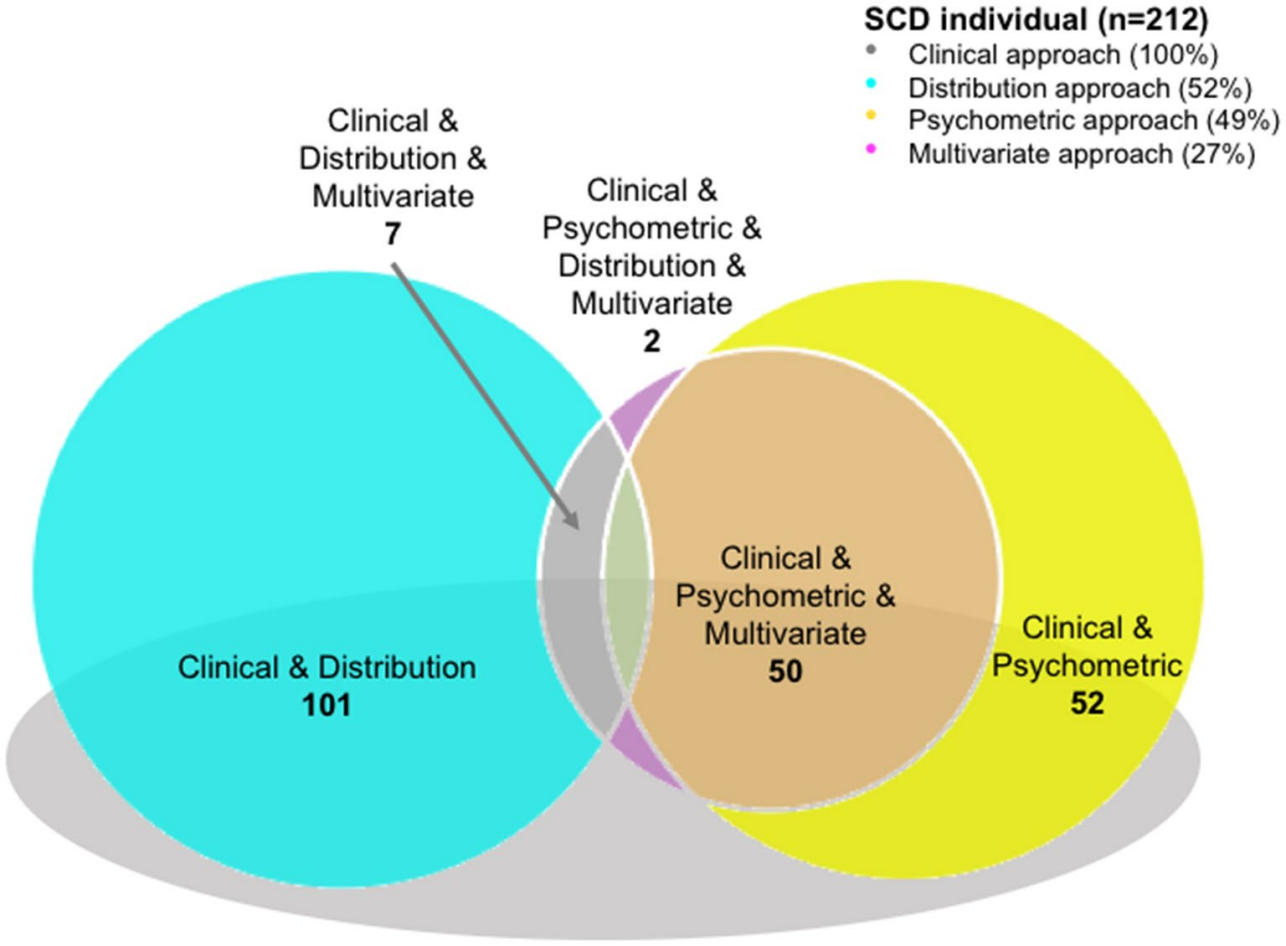
Overlap between the four SCD operationalization approaches. Percentage values indicate the frequency of individuals with subjective complaints classified as SCD by the different approaches and their combination.

Figure 3 illustrates the overlap between the four SCD operationalization approaches. The *Clinical* approach included all the SCD individuals and overlapped with the other three. Two clusters can be identified going from higher sensitivity (overlap with the *Distribution* approach) to higher specificity (overlap with the *Psychometric* and *Multivariate* approaches).

In the *Psychometric, Distribution,* and *Multivariate* approaches, 51% (n = 108), 34% (n = 72), and 72% (n = 153) of the individuals with subjective cognitive complaints were not classified as SCD (nonSCD), respectively. The characteristics of those nonSCD individuals in each approach are displayed on the supplementary Table S3 and Figure S1.

### Clinical characterization of the SCD groups

The SCD groups were characterized by conducting random forest classification models (SCD *vs.* HC). We included all the demographic, clinical, and cognitive measures as predictive variables in the random forest models. The most important variables from each model and the summary of the results are displayed in Table 4A. All the models provided a classification error greater than chance, indicating that any combination of the variables was able to discriminate between the SCD subtypes and HC. Therefore, SCD groups were comparable to the HC group in demographic, clinical, and cognitive variables, as expected congruent with the definition of SCD (Table 4A). In addition, SCD groups in the *Clinical* and *Distribution* approaches were also comparable.

**Table 4:** Random forest results (classification and regression models).

**(A) Classifications models for demographic, clinical and cognitive variables**			
Model 1 *Clinical* approach	Model 2 *Psychometric* approach	Model 3 *Distribution* approach	Model 4 *Multivariate* approach
Depression VR Delayed Recall PCV Motor reaction time Age Stroop 1 Sheet	Depression VR Delayed Recall Age Stroop 2 sheet Luria's—Left alternative movement	VR Delayed recall Depression PCV Motor recation time Age Stroop 1 sheet	Depression Age VR Delayed Recall Stroop 1 Sheet AVLT-Learning Trial
N = 399	N = 291	N = 327	N = 246
Error by chance = 80%	Error by chance = 50%	Error by chance = 80%	Error by chance = 50%
*Classification error:* HC (n = 187): 5.4% aSCD-sd (n = 40): 100% aSCD-md (n = 67): 77.6% naSCD-sd (n = 68): 100% naSCD-md (n = 37): 100%	*Classification error:* HC (n = 187): 14.1% SCD-90thPC (n = 104): 56.7%	*Classification error*: HC (n = 187): 0% amSCD (n = 40): 100% anSCD (n = 52): 100% am-anSCD (n = 29): 100% atSCD (n = 19): 100%	*Classification error:* HC (n = 187): 2.2% SCD-multivariate (n = 59): 71.2%

**(B) Regression models for the AD signature atrophy pattern and the WMSA load**			
Model 1 AD signature atrophy (R^2^ = 0.1)		Model 2 WMSA load (R^2^ = 0.3)	
Predictive variables	*Imp*	Predictive variables	*Imp*
Sex	27.0	Age	26.0
Memory complaint	17.4	ICV	12.9
Word-finding complaint	15.3	Sex	6.3
Orientation complaint	12.2	Writing complaint	4.6
Reading complaint	5.7	Word–finding complaint	3.8
Facial recognition complaint	–	Reading complaint	–
Language comprenhension complaint	–	Memory complaint	–
Writing complaint	–	Language comprenhension complaint	–
WAIS-III Information subtest	–	Orientation complaint	–
Language production complaint	–	WAIS–III Information subtest	–
Executive functions complaint	–	Facial recognition complaint	–
		Executive function complaint	–

In random forest classification models **(A)**, the five most important variables and a summary of the classificatory performance is reported for each operationalization approach. The classification error needs to be lower than the expected error by chance for a model to be reliable. In random forest regression models **(B)**, importance (*Imp*) is displayed for those predictive variables that reached values above zero. Age and ICV were included to investigate their contribution to the prediction of the WMSA load, but not to the prediction of the AD signature atrophy pattern because this variable was adjusted by age and ICV during the creation of this index.

*HC* healthy controls, *aSCD-sd* amnestic Subjective Cognitive Decline-single domain, *aSCD-md* amnestic Subjective Cognitive Decline amnestic-multiple domain, *naSCD-sd* non-amnestic Subjective Cognitive Decline-single domain, *naSCD-md* non-amnestic Subjective Cognitive Decline-multiple domain, *SCD-90thPC* Subjective Cognitive Decline defined by the presence of two or more cognitive complaints = corresponding to the 90th PC of the total amount of cognitive complaints variable, *anSCD* anomic Subjective Cognitive Decline, *amSCD* amnestic Subjective Cognitive Decline, *am-anSCD* amnestic and anomic Subjective Cognitive Decline, *atSCD* atypical Subjective Cognitive Decline, *SCD-multivariate* Subjective Cognitive Decline defined by the presence of language production = language comprehension and/or writing alone or in combination with other complaints, *AD* Alzheimer's disease, *WMSA* White Matter Signal Abnormalities, *Imp* Importance, *ICV* intracranial volume, *WAIS-III* Wechsler Adult Intelligence Scale-3rd Edition.

**Figure 4:**
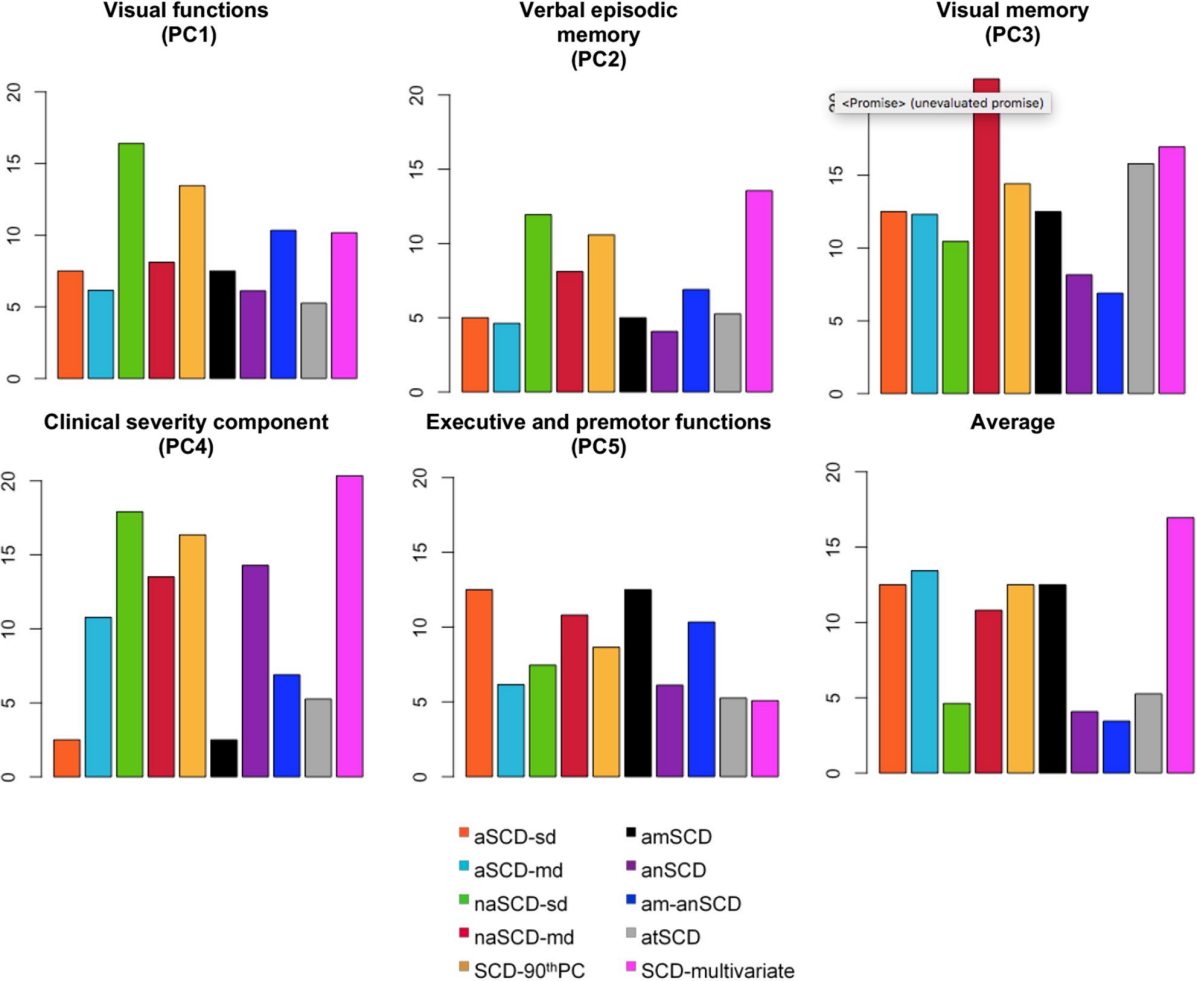
Cognitive profile of the SCD groups—Borderline performance. Percentage of SCD individuals with cognitive performance below the 10th percentile is reported for each SCD operationalization approach and subtype. The y-axis shows the percentage of SCD individuals below the 10th percentile. Higher percentage indicates that more individuals in a given group have borderline performance. This analysis was conducted only using SCD data. All the scores were previously adjusted for age, sex, and the WAIS-III Information subtest using multiple linear regression. The five components obtained in the PCA (Principal Component Analysis) were selected for this analysis. aSCD-sd, amnestic Subjective Cognitive Decline—single domain; aSCD-md, amnestic Subjective Cognitive Decline—multiple domain; naSCD-sd, non-amnestic Subjective Cognitive Decline—single domain; naSCD-md, non-amnestic Subjective Cognitive Decline—multiple domain; SCD-90thPC, Subjective Cognitive Decline defined by the presence of two or more cognitive complaints, corresponding to the 90thPC of the total number of complaints variable; anSCD, anomic Subjective Cognitive Decline; amSCD, amnestic Subjective Cognitive Decline; am-anSCD, amnestic and anomic Subjective Cognitive Decline; atSCD, atypical Subjective Cognitive Decline; SCD-multivariate, Subjective Cognitive Decline defined by the presence of language production, language comprehension and/or writing complaints alone or in combination with other complaints.

Once demonstrated that there is no objective cognitive impairment in the SCD individuals, we then investigated borderline performance (SCD individuals falling below the 10th percentile of cognitive performance). For simplicity, we investigated the five components of the PCA instead of the 67 cognitive variables (Fig. 4). Visual inspection of Fig. 4 shows that the *Multivariate* approach identified individuals with worst performance in verbal episodic memory (PCA2) and, as expected, in component #4 (PCA4). SCD subtypes endorsing memory complaints (i.e. aSCD-sd, aSCD-md, and amSCD) showed worst performance in executive and premotor functions (PCA5). The naSCD-md subtype (Clinical approach) showed worst performance in visual memory abilities (PCA3).

### AD signature atrophy and WMSA

**Figure 5:**
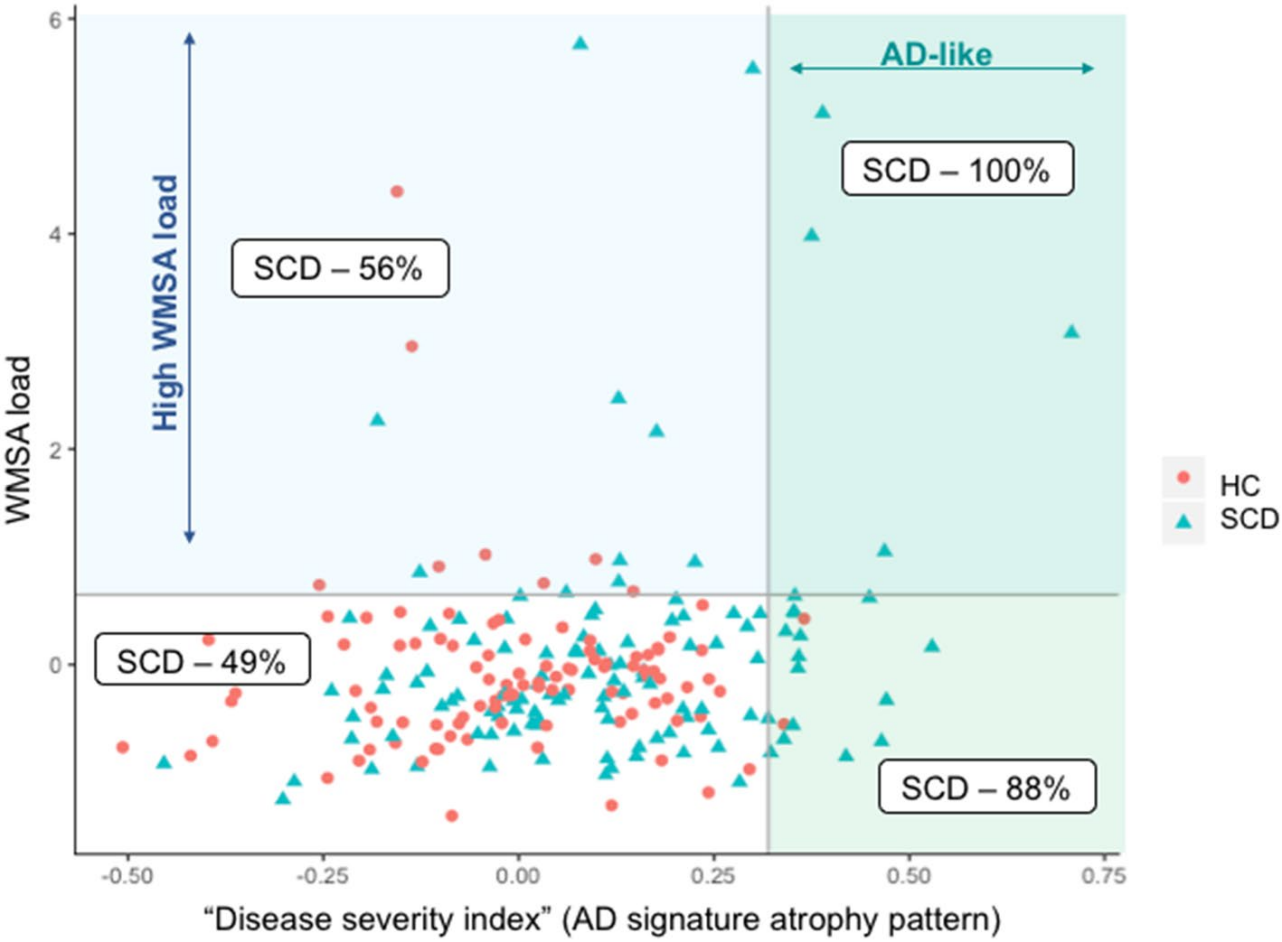
AD signature atrophy pattern versus WMSA load. The AD signature atrophy pattern and the WMSA load are treated as continuous variables in all the analyses in this study. Only for representation purposes in this figure, both measures were dichotomized to reflect an AD-like pattern of brain atrophy and high load of WMSA. This was done by using the 90th percentile cut-off as in previous studies^41^. The distribution of SCD and HC individuals (color legend) was plotted and, for each quadrant, the percentage of SCD individuals is reported. HC, healthy controls; SCD, subjective cognitive decline; WMSA, white matter signal abnormalities; AD, Alzheimer’s disease.

MRI data were available for 220 participants (55%). Compared with the HC, all operationalization approaches displayed increased disease severity index, reflecting an AD signature atrophy (Table 3). Regarding the WMSA (age and ICV included as covariates), the aSCD-md subtype (*Clinical* approach) showed significantly increased WMSA as compared with the HC (Table 3). A trend for a significant increase in WMSA (*p* = 0.074) was observed for the anSCD subtype (*Distribution* approach) (Table 3). Partial correlations were performed within each separate SCD group, controlled by age and ICV. Correlations showed no significant association between the AD signature atrophy and the WMSA load**.** Additionally, all individuals were classified as having normal/abnormal values in the AD signature atrophy pattern and the WMSA load based on the 90th percentile (Fig. 5). Interestingly, 100% of the individuals with abnormal values in both biomarkers were SCD individuals. Furthermore, 88% of the individuals with an abnormal AD signature atrophy pattern were SCD individuals, most showing an amnestic profile (aSCD-sd, aSCD-md, or amSCD). In contrast, 56% of the individuals with abnormal WMSA load were SCD individuals, and most showed a non-amnestic profile, predominantly anomic (naSCd-sd and anSCD). Regression random forest models were conducted including cognitive complaints, age, sex, depressive symptomatology, and ICV (only for WMSA) as predictors. Results showed that, in the whole sample, the AD signature pattern was mainly predicted by the memory complaint and sex, while WMSA were predicted by the writing complaint, age, ICV, and sex. The word-finding complaint also predicted both biomarkers, and complaints in orientation and reading marginally predicted the AD signature atrophy pattern. Model parameters are reported in Table 4B.

## Discussion

In the current study we addressed one of the priorities for SCD research at present, namely, gaining knowledge on the impact of different SCD operationalization approaches on the resulting SCD groups^3^. We tested four alternative approaches to operationalize SCD on a large community-based cohort. We then characterized the resulting SCD groups across a comprehensive set of demographical, clinical, and cognitive measures. We also sought to investigate potential AD and cerebrovascular underlying pathologies through surrogate sMRI biomarkers. We found that memory and word-finding were the most frequent complaints. However, they interrelated differently with the other complaints leading to an amnestic cluster strongly associated with AD, as well as to an anomic cluster, in which cerebrovascular disease also played a role. Writing, language comprehension, and language production complaints were also relevant, for example, delineating borderline performance on activities of daily living and verbal episodic memory.

The two first operationalization approaches were hypothesis-driven and were based on well-established MCI criteria. A high proportion of individuals endorsed two or more complaints (*Psychometric* criteria), similar to previous SCD studies investigating the amount of complaints^8^. Further, aSCD-md was one of the most frequent subtypes. Both findings support the notion of SCD as a multi-domain condition^42,43^, and highlight the heterogeneity within SCD. However, many previous studies have only recruited amnestic forms due to their focus on AD^5^. In addition, previous studies have usually included more homogeneous samples than that of the current study, because they were based on clinical settings where the frequency of memory complaints is higher^1,30,42–45^. In line with our results, non-amnestic complaints are frequently reported when heterogeneous community or population-based cohorts are investigated^5–7^. In particular, the word-finding complaint is frequently reported^6,7^. In the current study, we demonstrated that the *Distribution* operationalization approach translated these findings directly to SCD subtypes, providing a classification that could be useful in community-based cohorts as compared with operationalization approaches influenced by memory complaints (*Clinical* and *Psychometr*ic approaches).

In our fourth operationalization approach we used a data-driven method to identify the most clinically relevant cognitive variables based on their interrelation with measures of activities of daily living. Of interest, complaints that better predicted these cognitive and clinical variables included writing, language production, and language comprehension, but not memory. Our interpretation of this finding is that memory and/or word-finding complaints may be present in SCD individuals with an underlying neurodegenerative disorder^7,46–48^. However, since memory and/or word-finding complaints are very frequent in our cohort, rarer complaints such as writing, language production, and language comprehension seem to predict features of preclinical stages of a neurodegenerative disorder better (i.e. worse performance in measures of cognition and activities of daily living). Worse performance in these cognitive and clinical variables was also associated with lower crystallized intelligence/education (measured by WAIS-III Information subtest). This finding suggests that these complaints may reflect more premorbid cognitive or education status than preclinical changes associated to a neurodegenerative disease. However, below we discuss two other findings, i.e., cognitive profiles and sMRI biomarkers, that support the interpretation of these atypical complaints as predictors of features related to preclinical stages of a neurodegenerative disorder.

Writing, language production, and language comprehension complaints, which catalyze the *Multivariate* SCD group, were associated with subclinical impairment in verbal episodic memory (learning and recognition). These complaints, as well as memory complaints, were strongly associated with the AD signature atrophy pattern. Importantly, this AD signature atrophy pattern has been related to higher amyloid burden, higher frequency of the APOE ε4 allele, and greater progression to MCI and dementia in SCD individuals^31^. Thus, this specific cluster of complaints could be closely related to AD pathology. In contrast, word-finding complaints, which catalyze several of the non-amnestic subtypes, were associated with subclinical impairment in executive and premotor functions. There was also an association with memory functioning, since word-finding saturates on both the amnestic and non-amnestic clusters, but word-finding seemed to be more closely related with WMSA than with the AD signature atrophy. Previous studies have linked word-finding with non-AD pathologies^49^ and normal aging^50^ in addition to AD^7^. Therefore, we suggest that the anomic cluster of complaints in this study could be related to cerebrovascular or mixed pathology (AD plus cerebrovascular pathology). These results are consistent with MCI research showing that amnestic MCI is more closely associated with AD pathology, while non-amnestic and multi-domain MCI forms are more commonly related to cerebrovascular, mixed, or other pathologies^51^.

Some limitations should be mentioned. Our data is cross-sectional and from a single center. Future research should focus on validating our findings longitudinally and on independent cohorts. It is of relevance to ascertain which is the best operationalization method for identifying SCD individuals at highest risk for developing cognitive decline in the future. Our current analyses should be extended to include direct biomarkers of amyloid and neurofibrillary tangle pathology. Nonetheless, we investigated a community-based cohort, where the a-priori prevalence of AD is much lower than in clinical cohorts, and cerebrovascular and age-related tauopathies are more prevalent.

## Conclusions

In conclusion, our findings highlight the distinction between amnestic/non-amnestic phenotypes in SCD, perhaps anticipating corresponding subtypes of MCI, and different AD presentations and other dementias. Based on our findings we suggest that the SCD operationalization approach needs to be chosen depending on several factors, including the aims of the study, the source of the individuals, the clinical purpose, the characteristics of the clinical center, and the target of the clinical trial, among others. If the aim is early detection of any neurodegenerative disorder, the *Clinical* approach seems to have greater sensitivity but lacks of specificity and should be combined with the *Multivariate* approach. However, this strategy may overlook anomic forms in community-based cohorts. Hence, the *Distribution* approach may be a good starting point to explore the characteristics of the cohort, and other approaches shall be used later on depending on the frequency of amnestic and anomic profiles. To our knowledge, the current study is the first in applying and comparing different SCD operationalization approaches in the same cohort. We provide relevant data that could be used as preliminary research guidance. Since SCD-I support certain flexibility in the classification of SCD^1,3^, individual studies may still vary in their major aims. Therefore, it is imminent that researchers clarify how they operationalize SCD and why they choose a given approach. Our study could serve as a preliminary framework to guide and support their decision. However, more validation work needs to be done to be able to directly generalize our methods and reach a standardized operational/diagnostic criterion for SCD. The field needs to move forward conducting large multi-center studies that investigate operationalization approaches of SCD in different cohorts and using various instruments for measuring subjective complaints. Our present study is only a preliminary step and we hope that our findings help pave the way and encourage continuing the task of standardizing the operationalization of SCD in the near future.

## Supplementary Information

Supplementary Information.
