# Supplementary material for: Comparing different approaches for operationalizing subjective cognitive decline: impact on syndromic and biomarker profiles
Source: Sci Rep. 2021 Feb 23;11:4356. doi: 10.1038/s41598-021-83428-1 (PMC7902653; doi:10.1038/s41598-021-83428-1)
Supplement: Supplementary file 1 — Supplementary Information. [file 41598_2021_83428_MOESM1_ESM.docx]

**Comparing different approaches for operationalizing Subjective Cognitive Decline:**

**impact on syndromic and biomarker profiles**

Diaz-Galvan, P; Ferreira, D; Cedres N; Falahati F; Hernández-Cabrera, J; Aimes, D; Barroso, J; Westman, E

| **Table S1** Correlations between the nine cognitive complaints and the demographic, clinical, cognitive and neuroimaging variables | | | | | | | | | |
| --- | --- | --- | --- | --- | --- | --- | --- | --- | --- |
|  | **Memory** | **Word-finding** | **Language production** | **Orientation** | **Face recognition** | **Language comprenhension** | **Executive functions** | **Reading** | **Writing** |
| *Age* | 0.11* | 0.25*** | 0.15** | 0.01 | 0.08 | 0.10 | 0.11* | 0.12* | 0.16** |
| *WAIS-III Information* | -0.08 | -0.06 | 0.00 | -0.03 | -0.05 | -0.09 | -0.15** | -0.10* | -0.11* |
| *Depressive sympt* | 0.26*** | 0.29*** | 0.16** | 0.17*** | 0.03 | 0.16** | 0.05 | 0.09 | 0.12* |
| *BDRS* | 0.14** | 0.20*** | 0.14** | 0.08 | 0.14** | 0.11* | 0.04 | 0.12* | 0.22*** |
| *FAQ* | 0.09 | 0.06 | 0.08 | 0.11* | 0.10 | 0.10 | 0.08 | -0.01 | -0.01 |
| *MMSE* | -0.04 | -0.14** | -0.06 | -0.04 | -0.03 | -0.10* | -0.06 | -0.05 | -0.11* |
| *PC1* | -0.08 | -0.11* | -0.06 | 0.02 | -0.11* | -0.07 | -0.14** | -0.08 | -0.05 |
| *PC2* | -0.08 | -0.03 | -0.09 | 0.01 | 0.03 | -0.04 | -0.01 | 0.01 | -0.06 |
| *PC3* | -0.02 | -0.13** | -0.08 | -0.13* | -0.15** | -0.07 | -0.07 | 0.05 | -0.05 |
| *PC4* | -0.05 | -0.05 | -0.03 | -0.05 | -0.06 | -0.09 | -0.10 | -0.05 | -0.16** |
| *PC5* | -0.01 | -0.09 | -0.08 | 0.00 | 0.00 | -0.05 | -0.03 | -0.04 | -0.04 |
| *AD signature atrophy* | 0.07 | 0.07 | 0.08 | 0.09 | 0.02 | 0.06 | 0.09 | 0.09 | -0.02 |
| *WMSA* | 0.07 | 0.11 | 0.07 | -0.05 | 0.06 | -0.10 | 0.01 | 0.11 | 0.14* |
| Spearman’s correlation coefficients are reported.  *p< 0.05  **p<0.01 ***p<0.001  Abbreviations: WAIS-III = Wechsler Adult Intelligence Scale - 3^rd^ Edition; MMSE = Mini Mental State Examination; FAQ = Functional Activity Questionnaire; BDRS = Blessed Dementia Rating Scale; : PC1 = visual functions; PC2 = Verbal episodic memory; PC3 = visual memory; PC4 = clinical severity component; PC5 = executive and premotor functions; AD = Alzheimer’s disease; WMSA = white matter signal abnormalities. | | | | | | | | | |

| **Table S2** Characteristics of the individuals with subjective cognitive complaints not classified by the *psychometric, distribution* and *multivariate* approaches | | | | | | | | | | | | | |
| --- | --- | --- | --- | --- | --- | --- | --- | --- | --- | --- | --- | --- | --- |
|  | HC (n=187) | *Pyshometric* approach | | | *Distribution* approach | | | | | | *Multivariate* approach | | |
|  |  | SCD-90thPC (n=104) | **nonSCD**  **(n=108)** | *p* | amSCD  (n=40) | anSCD  (n=52) | am-anSCD (n=29) | atSCD (n=19) | **nonSCD (n=72)** | *p* | SCD-multivariate (n=59) | **nonSCD (n=153)** | *p* |
| Age, y | 55.66 (11.3) | 62.37 (11.2)^a^ | 58.57 (11.0) | <0.001 | 57.38 (11.5)^b^ | 60.02 (10.4) | 58.59 (10.6) | 57.05 (12.0) | 64.08 (11.0)^a^ | <0.001 | 61.93 (11.9)^a,b^ | 58.99 (11.0)^a^ | <0.001 |
| Sex, % females | 47 | 63^a^ | 60^a^ | 0.01 | 60 | 60 | 55 | 63 | 67 | 0.073 | 67^a^ | 62^a^ | <0.001 |
| WAIS-III, Information subtest | 16.27 (6.3) | 15.04 (6.3) | 14.85 (5.9) | 0.104 | 14.73 (6.1) | 15.14 (5.7) | 16.35 (6.3) | 13.84 (12.0) | 14.65 (6.3) | 0.511 | 14.49 (6.3) | 15.12 (6.0) | 0.439 |
| MMSE | 28.73 (1.3) | 28.46 (1.2) | 28.78 (1.2) | 0.131 | 28.95 (1.2) | 28.61 (1.2) | 26.9 (0.9) | 28.89 (1.2) | 28.26 (1.3) | 0.416 | 28.43 (1.3) | 28.75 (1.2) | 0.290 |
| FAQ | 0.28 (0.7) | 0.28 (1.0) | 0.31 (0.6) | 0.073 | 0.30 (0.6) | 0.32 (0.7) | 0.14 (0.4) | 0.42 (0.1) | 0.58 (1.0)^a^ | 0.042 | 0.54 (1.0)^a,b^ | 0.31 (0.7) | 0.016 |
| BDRS | 0.43 (0.8) | 0.99 (1.1)^a,b^ | 0.6 (0.8) | <0.001 | 0.51 (0.8)^b^ | 0.66 (0.9) | 0.62 (1.0) | 0.84 (1.0) | 1.1 (1.1)^a^ | <0.001 | 1.09 (1.1)^a,b^ | 0.62 (0.9)^a^ | <0.001 |
| Depressive sympt. | -0.54  (-1.3-3.0) | 0.34  (-1.0-3.9)^a^ | -0.10  (-1.3-3.00)^a^ | <0.001 | -0.1  (-1.3-2.1)^a^ | -0.1  (-1.3-3.0)^a^ | 0.34  (-0.8-2.1)^a^ | -0.1  (-1.0-3.5)^a^ | 0.34  (-1.0-3.9)^a^ | <0.001 | 0.34  (-1.0-3.9)^a^ | -0.10  (-1.3-3.9)^a^ | <0.001 |
| AD signature atrophy, (0-1) | -0.01 (0.2) | 0.07 (0.2)^a,b^ | 0.14 (0.2)^a^ | <0.001 | 0.14 (0.2)^a^ | 0.11 (0.2) | 0.04 (0.4) | 0.23 (0.2)^a^ | 0.09 (0.2) | <0.001 | 0.12 (0.2)^a^ | 0.11 (0.2)^a^ | <0.001 |
| AD signature atrophy, % AD-like | 2 | 12 | 20 | 0.016 | 33 | 9 | 6 | 23 | 17 | 0.189 | 15^a^ | 17^a^ | 0.016 |
| WMSA | 2621.3 (1207.0) | 2949.5 (2088.7) | 2300.7 (2167.0) | 0.100 | 2820.8 (982.9) | 3070.6 (1913.5) | 3023.0 (2353.9) | 3498.6 (2220.6) | 2660.8 (2347.6) | 0.704 | 2874.2 (1580.9) | 2465.5 (2473.4) | 0.421 |
| Values are reported as mean (SD) except for depressive symptomatology, where median (minimum and maximum values) are reported.  ^a^Significant differences with HC ^c^ n=220.  ^b^Significant differences with nonSCD.  ^c^ n=220.  ^d^AD signature atrophy pattern determined by the predictive OPLS index which range from 0 (HC-like pattern of atrophy) to 1 (Ad-like pattern of atrophy). Individuals were classified as AD-like when obtain abnormal values in this index, corresponding to 0.32 according to the 90^th^PC.  Abbreviations: HC = healthy controls; SCD-90thPC = Subjective Cognitive Decline defined by the presence of two or more cognitive complaints, corresponding to the 90^th^ PC of the total amount of cognitive complaints variable; anSCD = anomic Subjective Cognitive Decline; amSCD *=* amnestic Subjective Cognitive Decline; am-anSCD *=* amnestic and anomic Subjective Cognitive Decline; atSCD *=* atypical Subjective Cognitive Decline; SCD-multivariate = Subjective Cognitive Decline defined by the presence of language production, language comprehension and/or writing complaints, alone or in combination with other complaints; nonSCD = individuals with subjective cognitive complaints who were unclassified by the psychometric, distribution, and multivariate approaches; WAIS-III = Wechsler Adult Intelligence Scale - 3^rd^ Edition; MMSE = Mini Mental State Examination; FAQ = Functional Activity Questionnaire; BDRS = Blessed Dementia Rating Scale; sMRI = structural Magnetic Resonance Imaging; AD = Alzheimer’s disease; WMSA = white matter signal abnormalities. | | | | | | | | | | | | | |

**Figure S1**

| A |
| --- |
| 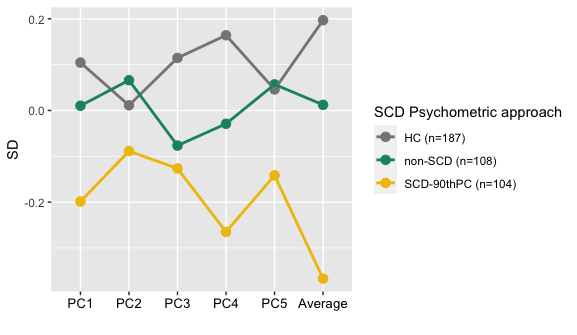 |
| B |
| 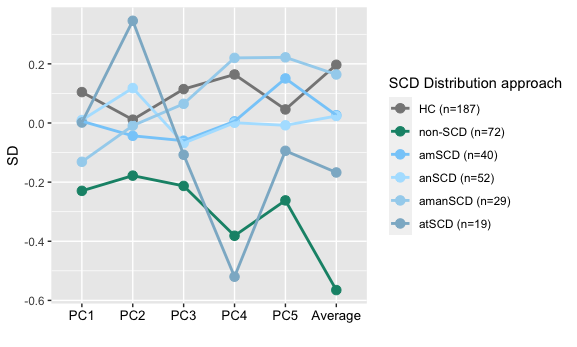 |
| C |
| 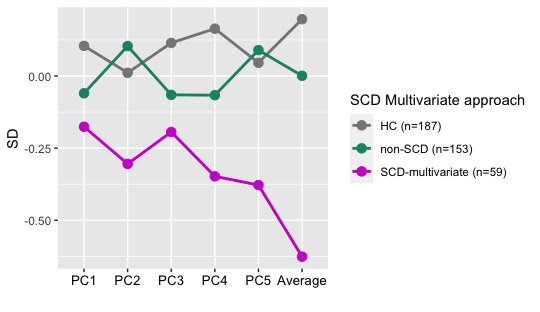 |

**CAPTION**

**Figure S1 Cognitive profile of the HC, SCD subtypes and SCD individuals non classified by the *Psychometric, Distribution*, and *Multivariate* approaches– Borderline performance.** The cognitive performance is reported for each SCD operationalization approach and subtype. The y-axis shows standardized scores on the five components obtained in the PCA (Principal Component Analysis) were selected for this analysis: PC1 = visual functions; PC2 = Verbal episodic memory; PC3 = visual memory; PC4 = clinical severity component; PC5 = executive and premotor functions.

**SUPPLEMENTARY METHODS**

**Additional Information on the “Disease severity index” (AD signature atrophy pattern)**

Below we explain the procedure to obtain the “disease severity index”, which reflects the AD signature atrophy pattern. This procedure is also fully detailed in a previous publication [1]. Briefly, an external training dataset including healthy controls without subjective cognitive complaints (HC) and Alzheimer’s disease (AD) patients is used to train a model for the discrimination between these two groups (i.e. classification model). Structural Magnetic Resonance Imaging (sMRI) data is used as input data. Subsequently, another dataset can be used as unseen data (in the current study we used the GENIC data). The unseen data is projected onto the classification model. By doing this, all the GENIC individuals receive a figure known as the “disease severity index”. This figure reflects the AD signature atrophy pattern, where values close to 0 have a HC-like pattern and values close to 1 have an AD-like pattern of brain atrophy. The index obtained can be used as a continuous variable, or also as a dichotomous variable by applying a certain threshold (i.e. diagnostic index: normal *vs.* abnormal).

***Training dataset***

The AIBL cohort (Australian Imaging Biomarkers and Lifestyle flagship study of ageing) was used as the training dataset. A full description of participants recruitment, including selection and diagnostic criteria, is published in Ellis et al. ^6^. We used the same training model than in our previous publication ^5^. In the current study, the two groups included in the discrimination model were the healthy controls without subjective cognitive complaints (n = 69) and the AD patients (n = 39) (**Table 1a)**.

| **Table 1. Demographics of the cohorts included in the study** | | | | | |
| --- | --- | --- | --- | --- | --- |
|  | **(a) AIBL cohort** | | | **(b) GENIC cohort (n=220)** | **AIBL *vs.* GENIC**  **p-value** |
|  | **Total sample (n=108)** | **HC (n=69)** | **AD (n=39)** |  |  |
| **Age** | 72.6 (7.7) | 72.7 (7.2) | 72.6 (8.7) | 54.8 (10.2) | <.001 |
| **Sex, % female** | 58 | 60 | 51 | 55 | .825 |
| **ICV** | 1540764 (153232) | 1551010 (161691) | 1522638  (137124) | 1452350  (161112) | <.001 |

Abbreviation: ICV, intracranial volume

***Unseen dataset (i.e. GENIC)***

The unseen data to project onto the classification model was the GENIC cohort described in the main manuscript. **Table 1b** shows the descriptive data of this GENIC cohort.

***Exploring potential confounders previous to projecting the unseen data***

Prior to projecting the unseen data, differences between training (AIBL cohort) and unseen (GENIC cohort) datasets were explored in variables known to influence brain morphology, i.e. age, sex, and total intracranial volume (ICV) (**Table 1**). We found that individuals from AIBL were significantly older and had increased ICV than participants from GENIC. No significant differences were found for sex.

***Input data for the classification model***

The 34 measures of cortical thickness from both hemispheres (68 in total), and the 21 measures of subcortical volume listed in **Supplementary Table 3** were used as input data for the classification model. However, due to the findings reported in the previous section, all these structural MRI measures were corrected by age and ICV before running the model. Briefly, the variance of each structural MRI measure explained by age and ICV was estimated using multiple linear regression. The estimation of the age effect was based only on the HC group, but the correction was done on both the HC and the AD groups. The reason for this is removing age-related changes while keeping AD-related changes on the AD group ^7^. The estimation of the ICV effect was based on both the HC and the AD groups, and the correction was applied to both. This age- and ICV-corrected MRI data was used in the subsequent analyses to obtain the index.

***Classification model***

The classification model was performed using the OPLS (Orthogonal Partial Least Square) method included in the software package SIMCA (Sartorius Stedim AB, Umeå, Sweden). The OPLS method separates the systematic variation in the data into two blocks: predictive and orthogonal. The first component of the model is predictive and includes information related to class separation (e.g. AD *vs.* HC). The orthogonal components in the model, if any, are related to other variation in the data not related to the actual problem, such as within class variation. Each model receives an R^2^(X), an R^2^(Y), and a Q^2^(Y) value, being Y referred to criterion variable and X to predictor variables. R^2^(X) represents the explained variance between Y and X, for the predicted and the orthogonal components. R^2^(Y) represents the model goodness of fit and refers to the fraction of the Y variation modeled in the component, using the predicted model. Q^2^(Y) defines how well the model predicts new data. The significance of a model is based on the Q^2^(Y) parameter and is reported as acceptable (Q^2^>0.1), good (Q^2^>0.5), and optimal (Q^2^>0.9) ^8^.

In this study, an OPLS classification model was trained on the AIBL cohort as explained above. A 7-fold cross validation method was conducted to separate AD patients (n=39) from HC (n=69). The age- and ICV-corrected structural MRI measures were used as input data to build the model. The model achieved an R^2^(X) value of 0.171, an R^2^(Y) value of 0.848, and a Q^2^(Y) value of 0.70, indicating a high performance to discriminate between the HC and AD groups. The brain regions that contributed the most to this model were the hippocampal volume, the precuneus, the right supramarginal gyrus, and the inferior parietal gyrus, all of them displaying reduced values in the AD group. The inferior part of the lateral ventricles also was important, displaying larger volume in the AD group, as in previous publications [1].

***Projection of GENIC data onto the AIBL classification model (“disease severity index”).***

In this step, a predictive model was conducted by projecting the same age- and ICV-corrected structural MRI measures from the GENIC sample onto the AIBL classification model. By doing this, the OPLS model assigns a score to each GENIC participant corresponding to the “disease severity index” (i.e. how much the brain pattern of each GENIC participant resemble the pattern of an AD patient or a healthy control from the AIBL cohort). A score close to one represents an AD-like pattern of brain atrophy and a score close to zero represents a HC-like pattern.

***AD-like pattern of atrophy (dichotomous variable)***

In this step, the “disease severity index”, which is a continuous variable in nature, is dichotomized in order to serve as a diagnostic index. In this way, the index will describe if a GENIC individual has an AD-like or a HC-like pattern of brain atrophy. A value of 0.5 has previously been used in the AIBL cohort as the cut-off for defining an AD-like pattern of brain atrophy ^5^. However, a cut-off based on the own sample distribution has recently shown better prognostic performance than the arbitrary cut-off of 0.5 ^9^. In particular, a cut-off of -1 SD based on the distribution of the healthy controls allowed to predict progression from mild cognitive impairment to AD with high performance ^9^. Thus, we adjusted this cut-off to our GENIC data by applying the 90th percentile to be consistent with the cut-offs used in this manuscript for the cognitive variables and the definition of the *Psychometric* SCD group.

After applying the 90th percentile cut-off in the GENIC data, 22 individuals were classified as having an AD-like pattern of brain atrophy (i.e. they displayed an AD signature pattern of atrophy).

The “disease severity index” referred in the previous section (continuous variable) is the measure used in most of the analyses in the manuscript. The dichotomized AD signature pattern of atrophy was used mainly for displaying purposes in the Figure 4 of the main manuscript.

***References***

1. Farias, S. T. *et al.* The measurement of everyday cognition (ECog): scale development and psychometric properties. *Neuropsychology* **22**, 531–44 (2008).

2. Rami, L. *et al.* The Subjective Cognitive Decline Questionnaire (SCD-Q): a validation study. *J. Alzheimers. Dis.* **41**, 453–66 (2014).

3. Valech, N. *et al.* Executive and language subjective dognitive decline complaints discriminate preclinical Alzheimer’s disease from normal aging. *J. Alzheimers. Dis.* **61**, 689–703 (2018).

4. Lezak, M. D., Howieson, D., Bigler, E. & Tranel, D. *Neuropsicological Assessment*. *Oxford University Press, Inc. 198 Madison Avenue, New York, New York 10016* (Oxford University Press, Inc., 2012).

5. Ferreira, D. *et al.* A ‘Disease Severity Index’ to identify individuals with Subjective Memory Decline that will progress to mild cognitive impairment or dementia. *Sci. Rep.* **7**, 188 (2017).

6. Ellis, K. A. *et al.* The Australian Imaging, Biomarkers and Lifestyle (AIBL) study of aging: methodology and baseline characteristics of 1112 individuals recruited for a longitudinal study of Alzheimer’s disease. *Int. Psychogeriatr.* **21**, 672–687 (2009).

7. Falahati, F. *et al.* The Effect of Age Correction on Multivariate Classification in Alzheimer’s Disease, with a Focus on the Characteristics of Incorrectly and Correctly Classified Subjects. *Brain Topogr.* **29**, 296–307 (2016).

8. Eriksson, L., Byrne, T., Johansson, E., Trygg, J. & Vikström, C. *Multi- and kegavariate data analysis: basic principles and applications*. (Umetrics academy, 2013).

9. Falahati, F. *et al.* Monitoring disease progression in mild cognitive impairment: Associations between atrophy patterns, cognition, APOE and amyloid. *NeuroImage Clin.* **16**, 418–428 (2017).
